# Successful reimplantation of extruded bone segment in lower limb open fractures: case report and literature review

**DOI:** 10.3389/fped.2024.1333575

**Published:** 2024-02-15

**Authors:** Xiongke Hu, Qian Tan, Guanghui Zhu, Kun Liu

**Affiliations:** Department of Pediatric Orthopedics, Hunan Provincial Key Laboratory of Pediatric Orthopedics, Hunan Children’s Hospital, Changsha, Hunan, China

**Keywords:** open fracture, bone extrusion, femoral fracture, reimplantation, ethylene

## Abstract

**Objective:**

The aim of this study is to summarize and demonstrate the different sterilization methods and surgical techniques for open fractures with impacted bone segments in the lower limbs.

**Methods:**

A retrospective analysis was conducted on the clinical characteristics, treatment methods, and outcomes of a case involving a 10.5 cm extruded segment of the femur in a 9-year-old male with a right femoral comminuted fracture treated at our center. Additionally, a retrospective review and summary were conducted on all reported cases of open fractures with impacted bone segments in the lower limbs.

**Results:**

Our center treated a 9-year and 11-month-old male child who presented with a Gustilo type IIIB open fracture of the femur along with a large segment of the femur being ejected as a result of a car accident. The child was resuscitated to correct hypovolemic shock, underwent emergency wound debridement, and had Ilizarov external fixation of the femur. The ejected femur segment was sterilized using ethylene oxide and re-implanted four days after the injury. A literature review showed that out of the cases of open fractures with impacted bone segments in the lower limbs, there were 14 cases involving the femur and 5 cases involving the tibia. Among them, sterilization was performed using povidone-iodine in 6 cases, high-pressure steam sterilization in 3 cases, and other methods including gamma-ray irradiation and soaking in antibacterial solution were used in the remaining cases. In terms of surgical methods, 7 cases were fixed with locking plates, 3 cases were fixed with external fixation devices, 1 case was immobilized in a cast, 1 case was fixed with an intramedullary rod, and 4 cases involved a combination of external fixation and internal fixation. The average time for re-implantation was 7.6 days after the injury. There were no serious complications such as infection or non-union observed in any of the cases during follow-up.

**Conclusion:**

Ethylene oxide can be considered a reliable choice for the reimplantation of displaced bone segments in open fractures after sterilization.

## Introduction

High-energy open fractures pose a challenging task in orthopedics due to their increased risk of infection and potential for delayed or nonunion healing. When traumatic compression leads to significant bone defects, the complexity of the condition is further exacerbated (1). In cases where the extruded bone fragments are lost, reconstruction can be considered using vascularized autogenous bone grafts or allografts (1, 2). However, when the extruded bone segment is retained, the challenge lies in its decontamination, sterilization, and timing of reimplantation. Open fractures with associated bone loss have a relatively low incidence, often classified as more severe Gustilo type III fractures. Prospective studies have found that the infection rate for Gustilo type IIIA open fractures is 45%, while the infection rate for Gustilo type IIIB fractures is 61% (3). Therefore, special attention is required for the sterilization of implanted bone grafts in order to reduce the infection rate. Currently, there is limited information available regarding the management of extruded bone segments. Different scholars have reported various cleaning methods such as high-pressure sterilization and 10% povidone-iodine soaking for the treatment of extruded bone segments (1, 4). However, there is still controversy regarding the sterilization methods, fixation techniques, and reimplantation procedures for extruded bone segments. In this case report, we present a successful reimplantation of an extruded segment of the femur in a 9-year-old boy using ethylene oxide sterilization. To our knowledge, this is the first reported case of successful reimplantation of an extruded bone segment using ethylene oxide sterilization. The patient provided consent for publication and presentation of the case data.

## Methods

We retrospectively reviewed a case involving a 9-year and 11-month-old male child who presented with a Gustilo type IIIB open fracture of the femur, along with a comminuted fracture of the right femur and a large segment of the femur being extruded, as a result of a car accident. The right femur experienced a high-energy injury, resulting in a predominant fracture in four segments, including the proximal segment of the femur, the free segment in the middle of the femur, the extruded segment of the femur, and the distal metaphyseal segment of the femur. Additionally, a 3 cm open wound is visible on the lateral aspect of the distal femur. The child was resuscitated to correct hypovolemic shock, underwent emergency wound debridement, and had Ilizarov external fixation of the femur. The extruded segment of the femur was sterilized using ethylene oxide before re-implantation.

A literature review was conducted using the keywords “Extruded,” “Femur,” “Tibia,” and “Reimplantation” to search the PubMed database for articles published up until October 2023.

### Case Introduction

**Figure 1:**
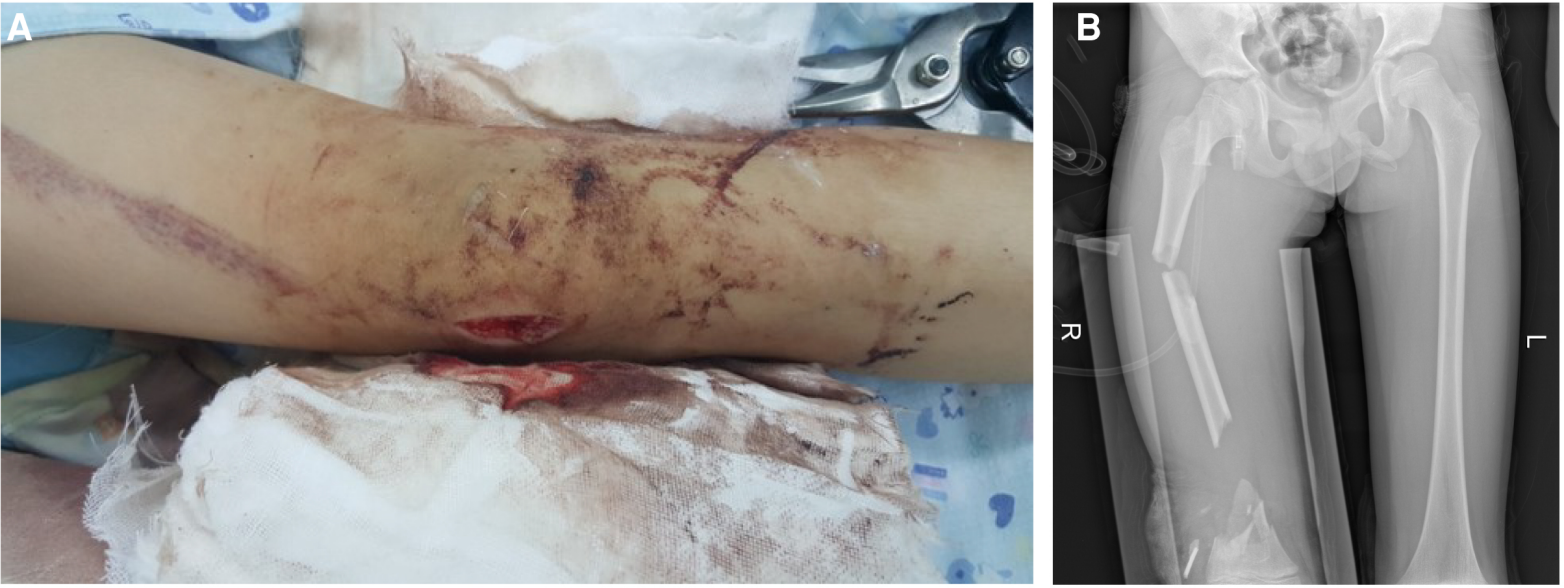
(**A**) A 4 cm open wound is visible at the distal end of the right femur. (**B**) Shows a plain radiograph of the femur, demonstrating a segmental fracture with significant bone loss in the right femoral diaphysis.

A 9-year-old boy sustained a right femoral Gustilo IIIB open fracture as a result of a collision between a motorcycle and a car. A approximately 3 cm wound was visible on the lateral aspect of the right thigh, accompanied by a 10.5 cm extruded segment of the femur. The fracture involved the midshaft of the right femur, with comminuted fracture at the distal end and extrusion of the femoral segment. The extruded femoral segment was not covered by periosteum. No significant nerve or vascular injury was identified in the right femur. The dorsalis pedis and posterior tibial pulses of the patient are palpable, and sensation in various areas of the lower leg is normal (Figure 1).

**Figure 2:**
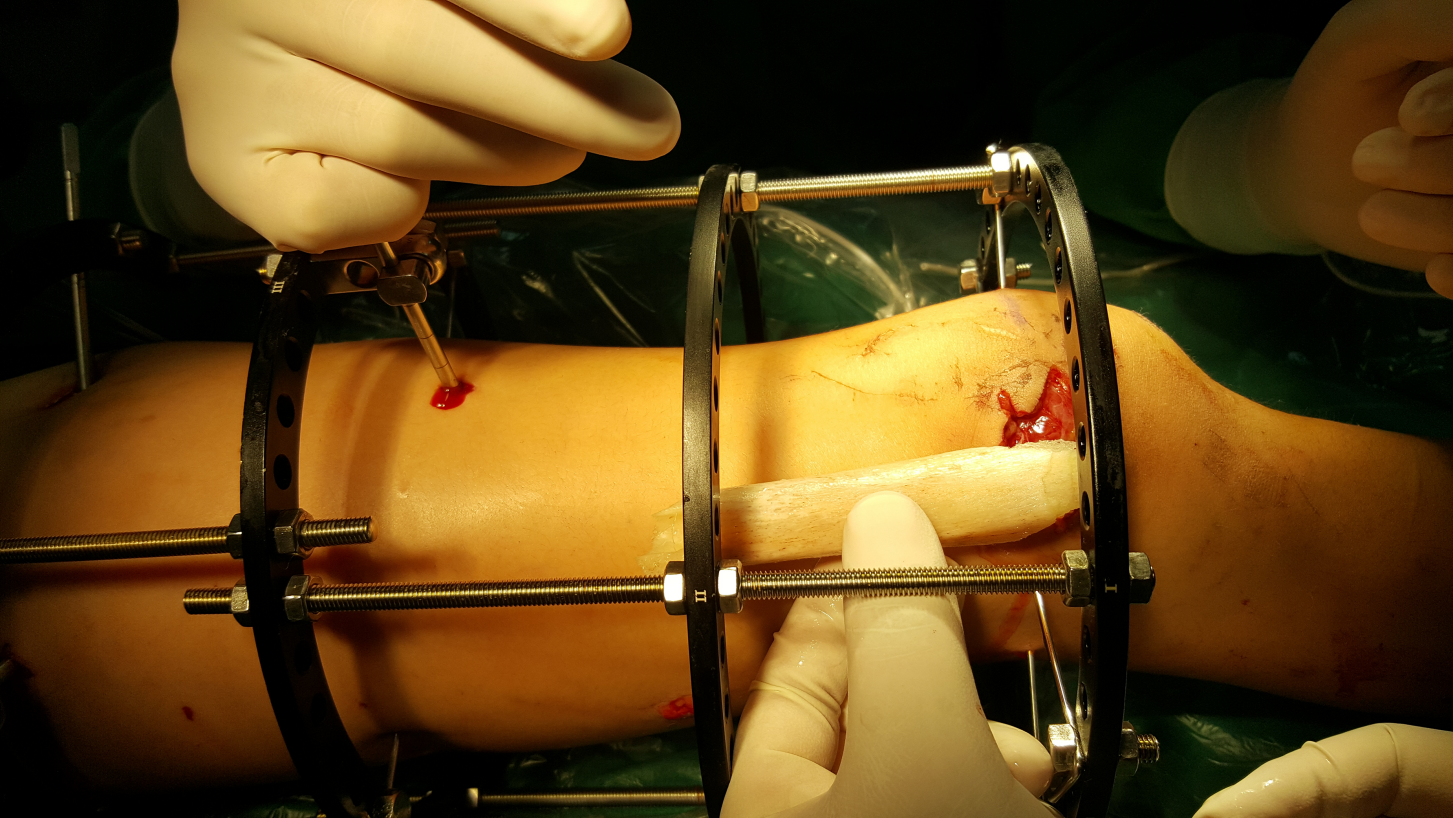
Ilustrates the emergency surgery performed on the patient to stabilize the fractured end of the femur, following correction of vital signs after injury.

The patient was transferred to our hospital from an external facility 12 h after the injury. After correcting the patient's blood loss and assessing vital signs, intravenous administration of cefazolin and tetanus toxoid was performed. The patient was taken to the operating room 3 h after admission. A copious pulsatile irrigation with normal saline and wound debridement was performed on the patient's right thigh through the open wound. The periosteum and soft tissue envelope surrounding the bone defect were well preserved. Subsequently, an Ilizarov external fixation frame was applied to stabilize the proximal, midshaft, and distal fragments of the femur. An external fixation ring was placed at the site of the extruded femoral segment for fixation purposes (Figure 2).

**Figure 3:**
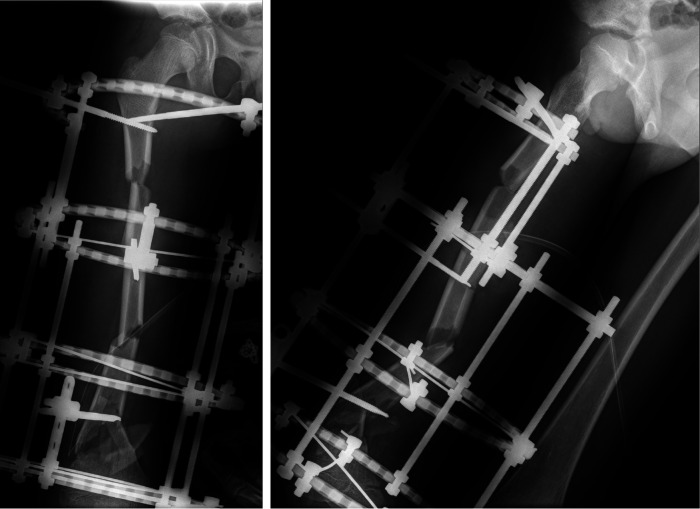
On the 5th postoperative day, shows a right lateral view x-ray of the femur, indicating a generally restored femoral alignment, but still presenting some degree of angular deformity.

Reconstruction of the extruded segment of the femur is crucial for the restoration of femoral structure. Firstly, we washed the 10.5 cm extruded femoral segment with a large amount of normal saline to remove severe contamination. Subsequently, the extruded femoral segment was sterilized using ethylene oxide, with a controlled concentration of 800–1,000 mg/L, temperature of 55–60°C, and relative humidity of 60%–80%, for a total sterilization time of 6 h. After sterilization, the extruded femoral segment was dissected within the sterilization cabinet to avoid residual ethylene oxide. On the 4th day of the initial surgery, the sterilized extruded femoral segment was reimplanted through the use of external fixation pins for stabilization (Figure 3).

**Figure 4:**
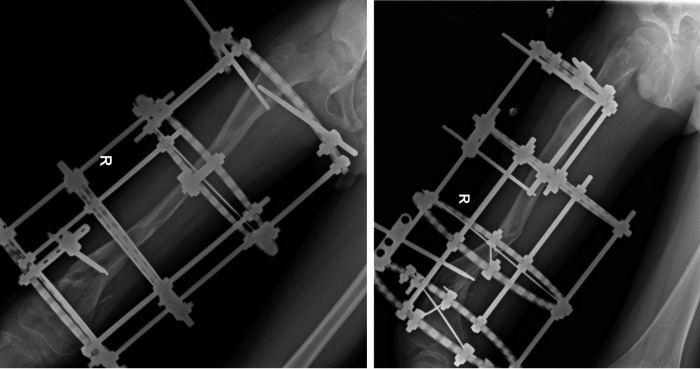
Taken 7 months postoperatively, shows a right lateral view x-ray of the femur, indicating fracture healing, followed by the removal of the external fixation device.

**Figure 5:**
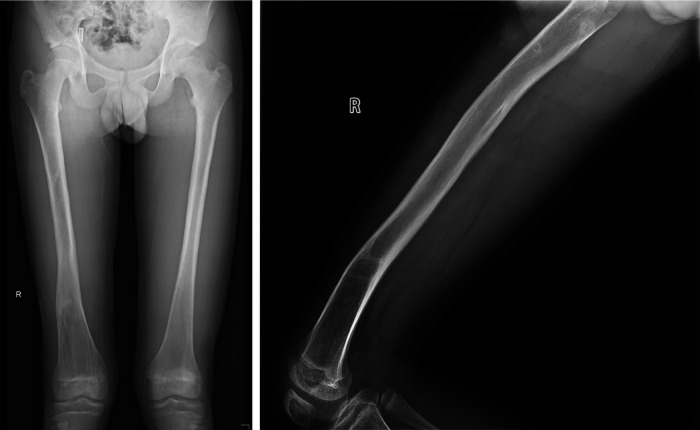
Taken during a 5-year follow-up examination, shows a right lateral view x-ray of the femur, demonstrating equal length of both femurs, good remodeling of the right femur, and slight bending seen on the lateral view.

**Figure 6:**
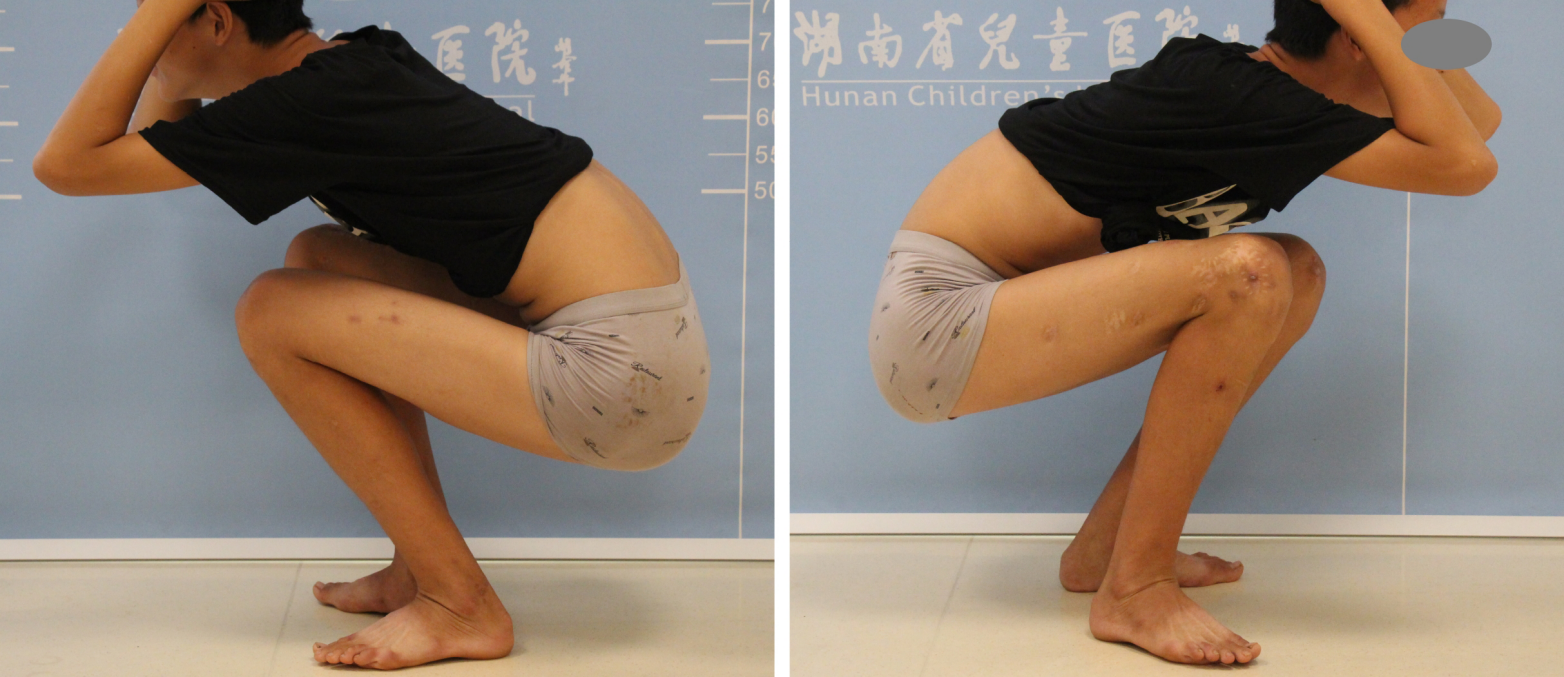
Taken during the 5-year follow-up, shows normal knee joint function.

During a follow-up examination 3 months postoperatively, formation of callus was observed at the fracture site. At 7 months postoperatively, the fracture line disappeared, and cortical bone continuity was achieved, leading to the removal of the external fixation device. At 18 months postoperatively, the femur had achieved bony union, albeit slightly more curved compared to the unaffected side. During a follow-up at 60 months postoperatively, the femurs were approximately equal in length, with slight local curvature observed in the right femur. The range of motion of the knee joint is 0°–135° (Figures 4–6).

Simultaneously, 19 cases of pediatric patients with open fractures and impacted bone segments in the lower limbs were also evaluated (Table 1). Among them, there were 14 cases involving the femur and 5 cases involving the tibia. Among the cases, 6 cases were sterilized using povidone-iodine, 3 cases were sterilized using high-pressure steam, and the remaining cases were treated with gamma-ray irradiation or soaking in antibacterial solution. In terms of surgical methods, 8 (42.1%) cases were fixed with locking plates, 3 (15.8%) cases were fixed with external fixation devices, 1 (5.3%) case was immobilized in a cast, 1 (5.3%) case was fixed with an intramedullary rod, and 4 (21.1%) cases involved a combination of external fixation and internal fixation. The average time for re-implantation was 7.6 days after the injury. There were no serious complications such as infection or non-union observed in any of the cases during follow-up.

**Table 1:** General information of children with open fractures and extruded bone Segments in lower limbs.

Cases	Gender/ Age (Y)	Location/Length (cm)	Sterilization methods	Surgical techniques	Replantation (D)
Hu et al.	M/9	Femur/10.5	Ethylene oxide	Ilizarov external fixator	4
Fansa et al. (1)	M/46	Femur/12	10% Povidone-Iodine soaking	Conversion from external fixation to locking plate	6
Abell (4)	M/26	Femur/19	Benzalkonium chloride soaking	–	7
Aizah et al. (5)	M/14	Femur/8	Gamma ray irradiation	Locking plate	14
Canovas et al. (6)	–/16	Tibia/12	boiling for 20 min	External fixator	Immediately
Farrelly et al. (7)	F/14	Tibia/15	1% Povidone-Iodine rinse	External fixator + locking plate	Immediately
Harper (8)	M/31	Femur/9	0.5% Neomycin soaking	External fixator + intramedullary rod	65
Kirkup et al. (9)	M/20	Femur/25	High-pressure steam sterilization after boiling	Internal fixation	12
Mazurek et al. (10)	M/15	Femur/13	4% Chlorhexidine Gluconate soaking	Locking plate	17
Meininger et al. (11)	M/33	Tibia/20	Saline flush	Intramedullary rod + screws	Immediately
Moosazadeh (12)	M/24	Femur/13.5	Gentamicin soaking	Locking plate	Immediately
Rouvillain et al. (13)	M/17	Femur/11	Autoclave sterilization	Locking plate	20
Shanmuganathan et al. (14)	M/30	Femur/10	Povidone-Iodine wash, Vancomycin soaking	Locking plate	Immediately
	M/62	Tibia/4.5	Povidone-Iodine wash, Vancomycin soaking	External fixator	Immediately
	M/18	Femur/10	High-pressure steam sterilization after Povidone-Iodine wash	Locking plate	Immediately
van Winkle er al. (15)	M/24	Femur/10	Bacitracin and Polymyxin B soaking	–	Immediately
Wu et al. (16)	M/16	Femur/14	Autoclave sterilization	External fixator + intramedullary rod	Immediately
Gannamani et al. (17)	M/45	Femur/18	Chlorhexidine wash, Vancomycin soaking	Locking plate	Immediately
Afshar et al. (3)	M/6	Tibia/5.5 + 4	10% Povidone-Iodine scrub, 2% Chlorhexidine soaking	Long leg cast immobilizatio	Immediately

## Discussion

Open fractures have always been a high-risk condition for infection due to factors such as the extent of soft tissue damage, wound cleanliness and irrigation methods, time of treatment post-injury, and antibiotic coverage (18). When open injuries are associated with an extruded bone segment, the situation becomes even more challenging. Adequate sterilization of the extruded bone segment, delayed replantation, stable fixation, and good patient health condition play critical roles in the decision-making process for replantation. Even with meticulous debridement and sterilization, re-implantation of an extruded bone segment exposed to severe contamination can still lead to devastating infections.

Currently, there are no specific guidelines for disinfection applied to the replantation of traumatic extruded bone segments. As early as 1965, Kirkup reported the successful replantation of a 25 cm extruded femoral segment using high-pressure boiling sterilization (10). Since then, several scholars have described their successful experiences with various sterilization techniques for extruded bone segments, including boiling, gamma-ray irradiation, high-pressure sterilization, and soaking in povidone-iodine, Betadine, and antibiotic solutions (1, 4–17, 19). Mat-Salleh et al. used discarded bone fragments from hip arthroplasty as samples and found that 0.5% chlorhexidine was significantly superior to povidone-iodine and alcohol in disinfecting contaminated bone (20). Some researchers analyzed the histomorphology of rat allografts after sterilization with high-pressure sterilization, gamma irradiation, and ethylene oxide and found no significant differences among the three groups (21). Mortazavi et al. (22) conducted a systematic review of the literature on contamination related to bone transplantation and found that the contamination rate of dislodged bone tissue during surgery was close to 40%. It was discovered that soaking the dislodged bone tissue in a 5% povidone-iodine solution for 10 min successfully removed the contamination and maintained cell viability. There have been some comparative studies on the effectiveness of different sterilization methods, but there is still a lack of unified standards. The choice of sterilization method also needs to consider the sterilization equipment commonly used in the medical facility.

High-pressure steam sterilization appears to have an advantage in eliminating pathogens, but some scholars also have concerns about the loss of graft bone activity and excessive loss of skeletal strength (23). Researchers used dry heat, high-pressure steam, ethylene oxide, and gamma radiation as four sterilization methods to treat dental scaffolds and found that all four methods achieved effective sterilization while preserving the molecular arrangement of the extracellular matrix (24). However, Zhou et al. studied the impact of gamma irradiation and ethylene oxide sterilization on the mechanical strength of cortical bone grafts and found that ethylene oxide sterilization was superior to gamma irradiation and better preserved the mechanical properties of cortical bone (25). Previous studies have indicated that ethylene oxide sterilization has no adverse effects on tendon biomechanical properties (26). Kurup et al. (27) collected cancellous bone washings from patients, which were sterilized using ethylene oxide and then used for treating benign bone lesions, non-unions, and other conditions. The low infection rate confirms that ethylene oxide can be a reliable choice for sterilizing allogeneic bone grafts and also offers good cost-effectiveness. We used ethylene oxide to sterilize the extruded bone segment before replantation. After a follow-up of 60 months, good bone fusion and no complications such as infection were observed. We believe that adequate sterilization and appropriate preservation of bone graft viability are both crucial. In cases where the surrounding soft tissues are intact but bone replantation is not feasible due to severe damage or contamination, options for reconstructing bone defects include allograft transplantation, guided membrane technology, or traction osteogenesis as alternative methods (28, 29).

There is no unified time frame for replanting extruded bone segments. The reported time ranges from immediate replantation to up to 65 days after trauma, with an average of 7.6 days. A retrospective study on open fractures of the limbs suggests that internal fixation should be performed as soon as general and local conditions have improved and infection is under control. The longer the delay in fixation, the higher the infection rate (30). The delay in replantation time is mainly dependent on the stability of the patient's vital signs and the contamination of the extruded bone segment. In our case, due to the child's traumatic shock after injury, Ilizarov external fixation was first performed to stabilize the fracture at both ends under the premise of stable vital signs, avoiding further displacement of the fracture. On the fourth day post-injury, after sterilization with ethylene oxide, the extruded bone segment was replanted and fixed with external fixation pins. There is no strict time restriction for replanting the extruded bone segment as long as the patient's vital signs are stable and inflammation is under control; generally, good bone fusion can be achieved. During the initial emergency surgery in our case, due to the incomplete nature of the femur, intramedullary fixation was omitted, resulting in slightly suboptimal alignment of the fractured femoral shaft. On the most recent follow-up, despite successful fracture remodeling, a residual posterior angulation of the midshaft femur was still observable on the lateral x-ray. Common surgical approaches for pediatric femoral shaft fractures include intramedullary nailing, external fixation, and a combination of both. Intramedullary nailing provides reliable central fixation and excellent resistance against bending forces, albeit with limited rotational stability. External fixation allows for effective control of rotational and shearing forces, though it exhibits relatively weaker resistance against bending forces and carries a slightly higher risk of complications. The combination of intramedullary nailing and external fixation combines the advantages of both fixation methods and has been proven to be superior in fracture alignment and early weight-bearing, although it requires a longer learning curve and surgical time (31).

## Conclusion

When facing the extrusion of a large bone segment, the management of each case should consider the replantation of the extruded bone segment on an individualized basis. When feasible, sterilization of the contaminated extruded bone segment with ethylene oxide can be considered. In cases involving open multiple fractures, Ilizarov external fixation is a good choice as it is associated with relatively smaller trauma and can stabilize multiple fractures.

## Data Availability

The original contributions presented in the study are included in the article/Supplementary Material, further inquiries can be directed to the corresponding author.
